# Facing the challenges of computational target prediction

**DOI:** 10.1186/1758-2946-6-S1-O3

**Published:** 2014-03-11

**Authors:** Karen T Schomburg, Matthias Rarey

**Affiliations:** 1 Center for Bioinformatics, University of Hamburg, Hamburg, Germany

## 

To which proteins does a compound bind? Is it selective or promiscuous? These are questions which can be answered by inverse virtual screening, which identifies potential targets for a molecule of interest. Experimental screening of a molecule on thousands of targets is costly and elaborate. Contrarily, structure-based computational methods are only limited by the availability of 3D structures, rendering them an important complement to experimental methods.

Our inverse screening method XxirT combines triangle descriptor matching [1] with a new ranking approach, considering a reference score for each pocket. A precalculated bitmap encoding of the descriptors and an efficient design of a database for 3D protein structures allows a rapid screening of thousands of protein-ligand complexes with a query compound.

Classical protein-ligand scoring functions are not capable of inter-target score comparison, since the absolute values are target-dependent. Therefore, in inverse virtual screening, a major problem is the design of a ranking scheme allowing the comparison of target scores with respect to one query compound [2]. A lack of available data for statistical evaluation of the ranking capability further complicates the task. Data sets mostly contain positive hits, e.g. binding affinities for one molecule to several proteins while lacking negative data points, such as ‘the molecule does not bind to this protein’. As a basis for statistical evaluation of our new inverse screening concept, we introduce a dataset consisting of a ligand set of approved drugs and the scPDB target database [3]. Drugs are well qualified for use in a method evaluation dataset, as they are well tested and had to pass selectivity standard tests to get approved. Therefore, their targets are comparably well-characterized, allowing a classification into true positives and true negatives. This approach is the first that evaluates a structure-based inverse screening method on a systematic statistical test.
